# Long-Lasting Myocardial and Skeletal Muscle Damage Evidenced by Serial CMR During the First Year in COVID-19 Patients From the First Wave

**DOI:** 10.3389/fcvm.2022.831580

**Published:** 2022-03-09

**Authors:** Laura Filippetti, Nathalie Pace, Jean-Sebastien Louis, Damien Mandry, François Goehringer, Maria-Soledad Rocher, Nicolas Jay, Christine Selton-Suty, Gabriela Hossu, Olivier Huttin, Pierre-Yves Marie

**Affiliations:** ^1^Department of Cardiology, CHRU-Nancy, Nancy, France; ^2^Université de Lorraine, INSERM, UMR-1254, Nancy, France; ^3^CHRU-Nancy, Université de Lorraine, CIC 1433, Nancy, France; ^4^Department of Radiology, CHRU-Nancy, Université de Lorraine, Nancy, France; ^5^Department of Infectious Diseases, CHRU-Nancy, Université de Lorraine, Nancy, France; ^6^Department of Medical Information, CHRU-Nancy, Université de Lorraine, Nancy, France; ^7^Université de Lorraine, INSERM, UMR-1116, Nancy, France; ^8^CHRU-Nancy, Université de Lorraine, Nuclear Medicine and Nancyclotep Platform, Nancy, France

**Keywords:** COVID-19, myocarditis, edema, skeletal muscle, cardiovascular magnetic resonance imaging

## Abstract

**Introduction:**

This observational CMR study aims to characterize left-ventricular (LV) damage, which may be specifically attributed to COVID-19 and is distant in time from the acute phase, through serial CMR performed during the first year in patients with no prior cardiac disease.

**Methods:**

This study included consecutive patients without any prior history of cardiac disease but with a peak troponin-Ic > 50 ng/ml at the time of the first COVID-wave. All had a CMR in the first months after the acute phase, and some had an additional CMR at the end of the first year to monitor LV function, remodeling, and abnormalities evocative of myositis and myocarditis - i.e., increased T1/T2 relaxation times, increased extracellular volume (ECV), and delayed contrast enhancement.

**Results:**

Nineteen consecutively admitted COVID-19 patients (17 men, median age 66 [57–71] years) were included. Eight (42%) had hypertension, six (32%) were obese, and 16 (84%) had suffered an acute respiratory distress syndrome. The 1^st^ CMR, recorded at a median 3.2 [interquartile range: 2.6–3.9] months from the troponin peak, showed (1) LV concentric remodeling in 12 patients (63%), (2) myocardial tissue abnormalities in 11 (58%), including 9 increased myocardial ECVs, and (3) 14 (74%) increased ECVs from shoulder skeletal muscles. The 2^nd^ CMR, obtained at 11.1 [11.0–11.7] months from the troponin peak in 13 patients, showed unchanged LV function and remodeling but a return to normal or below the normal range for all ECVs of the myocardium and skeletal muscles.

**Conclusion:**

Many patients with no history of cardiac disease but for whom an increase in blood troponin-Ic ascertained COVID-19 induced myocardial damage exhibited signs of persistent extracellular edema at a median 3-months from the troponin peak, affecting the myocardium and skeletal muscles, which resolved within a one-year time frame. Associations with long-COVID symptoms need to be investigated on a larger scale now.

**Clinical Trial Registration:**

NCT04753762 on the ClinicalTrials.gov site.

## Introduction

COVID-19 induced myocardial damage is complex and exhibits features consistent with inflammation and endothelium dysfunction, and thrombosis (1–3). It has been speculated that this myocardial damage might constitute a risk factor for developing heart failure, given the similarities in the profiles of patients at risk of heart failure with those of severe COVID-19 patients (4). There is, therefore, an urgent need to specify the nature of COVID-19 induced myocardial damage and investigate its impact over time.

Cardiac Magnetic Resonance (CMR) already documented myocardial tissue abnormalities at the acute or sub-acute phase of COVID-19 and, more specifically, increases in myocardial T1 and T2 relaxation times and an increased extracellular volume (ECV) (5, 6). This observation was at the time attributed to inflammatory edema. However, we do not yet know what the clinical consequences of these anomalies are in the long term and whether they correspond to a COVID-19 pathology specifically targeted to the heart or to a more diffuse edematous and inflammatory response (7). Myositis with skeletal muscle edema is also frequently observed during COVID-19 (8).

This observational CMR study aims to characterize the left-ventricular (LV) damage which may be specifically attributed to COVID-19 and distant in time from the acute phase, through serial CMR planned during the first year in patients with no previous history of cardiac disease but with significant increases in blood troponin-Ic during the initial COVID-19 hospitalization.

## Materials and Methods

### Patients and Study Design

The study included consecutive 18- to 80-year-old patients hospitalized in our Regional University Hospital for a COVID-19-related pathology between the 16^th^ and the 31^st^ of March 2020, which corresponded to the peak of the first COVID-19 wave in our region. Patients' COVID-19 status was ascertained by a positive reverse transcriptase-polymerase chain reaction test. Additional inclusion criteria were: (i) a peak troponin Ic > 50 ng/ml measured during hospitalization, (ii) the absence of any prior cardiac disease history, and (iii) health conditions required to endure the CMR-based monitoring which is currently prescribed for myocarditis patients in our center.

Baseline investigations were performed with the 1^st^ CMR within the first months following the acute phase and the follow-up investigations with the 2^nd^ CMR, at the end of the first year, on a 3T PRISMA Magnet (Siemens Medical Solutions, Erlangen, Germany). Echocardiography and blood analysis for routine biomarkers were also performed on the CMR days. Echocardiography data were obtained according to current recommendations (9, 10) with a General Electric^®^ device and the post-processing EchoPAC^®^ software.

### CMR Recording

The same CMR protocol was used in all patients and for both the 1^st^ and 2^nd^ CMR. LV function and remodeling were assessed on cine images recorded with a compressed sensing SSFP sequence (11), on contiguous short-axis slices and with the following parameters: 2 x 2 x 8 mm^3^ voxel size, 420 x 320 mm^2^ field of view (FOV), 60° flip angle (FA), 41 ms repetition time (TR), 2.9 ms interecho time, 1.27 ms echo time (TE), and 14 segments.

According to the “2018 updated Lake Louise Criteria” (12), signs of myocarditis were searched for (i) on longitudinal (T1) and transversal (T2) relaxation maps recorded with short-axis slices and, respectively, precontrast - Modified Look-Locker Inversion Recovery (MOLLI, acquisition scheme 5(3)3) and 2D TurboFlash sequences (12), and (ii) on contiguous late gadolinium enhancement images covering the LV on short-axis, vertical and horizontal long-axis directions with a fast multi-slice phase-sensitive inversion recovery sequence (13), 10 to 15 min after the injection of 0.1 mmol.kg^−1^ body weight of Dotarem^®^, (GUERBET, France). T1 maps were recorded with the following parameters: 1.4 x 1.4 x 8.0 mm^3^ voxel size, 371 x 278 mm^2^ FOV, 35° FA, 1 excitation, 180 ms time to inversion (TI), 267 ms TR, 1.11 ms TE, and 63 segments. For the T2 maps, these parameters were: 1.9 x 1.9 x 8.0 mm^3^ voxel size, 360 x 360 mm^2^ FOV, 12° FA, 201 ms TR, and 1.32/30/50 ms TE. For the LGE images, these parameters were: 2.1 x 2.1 x 8 mm^3^ voxel size, 400 x 380 mm^2^ FOV, 40° FA, 305 ms TI, 768 ms RT, 2.4 ms interecho time, 1.04 ms TE, and 1 excitation.

### CMR Analysis

**Figure 1:**
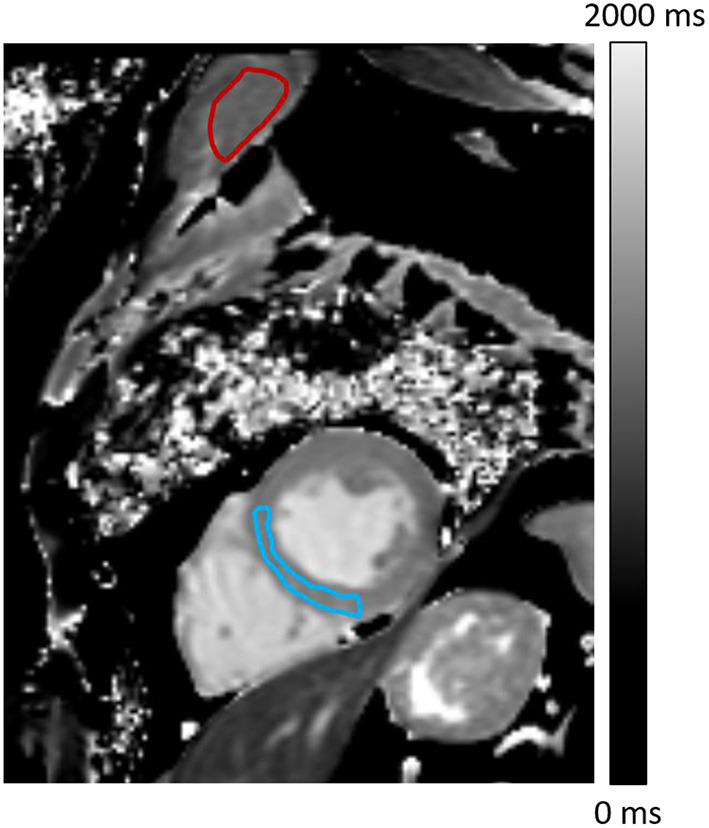
Pre-contrast T1 map showing regions-of-interest (ROIs) drawn to determine ECV and relaxation times on the myocardium (blue) and shoulders skeletal muscle (red).

CMR results were extracted with the Syngovia software (Siemens Medical Solutions, Erlangen, Germany), using a manual adjustment of the ventricular contours applied to determine LV mass, end-diastolic volumes, and ejection fractions (14). Ventricular volumes and LV mass were indexed to body surface area, and the LV mass/end-diastolic volume ratio was used to assess LV concentric remodeling (15, 16). Myocardial T1 and T2 were determined with regions of interest (ROI) drawn on a septal mid-ventricular area (Figure 1) (17). The myocardial extracellular volume (ECV), expressed as % myocardium volume, was conventionally computed from: (i) T1-pre values from the pre-contrast MOLLI sequence described above (ii) T1-post values from post-contrast MOLLI sequence (acquisition scheme: 4(1)3(1)2) acquired 10–15 min after the injection and (iii) individual hematocrit values (6, 12). The latter were obtained from blood sampled just before CMR, during placement of the intravenous catheter used for Dotarem^®^ injection.

The cardiac T1 maps were additionally used to determine the T1 and ECV of the shoulder skeletal muscles setting in the field of view (i.e., the pectoralis major, subscapularis, or infraspinatus) with careful exclusion of perimuscular fat and intramuscular tendons from the hand-drawn ROIs (Figure 1), as previously described (17).

### Criteria Used to Define Abnormal CMR Parameters

Normal limits for the main CMR parameters are summarized in Table 1. Most of these normal limits were derived from the 95% confidence intervals and obtained from local control populations with the same operator and extraction methods as the current COVID-19 study.

**Table 1:** Limits used to define abnormal values for the main CMR parameters obtained from local control populations with the same operator and extraction methods as the current COVID-19 study.

	**Women**	**Men**
Lower LV ejection fraction limits	51 %	49 %
Upper LV mass limits	57 g/m^2^	73 g/m^2^
Upper limits for LV mass/volume ratio	0.90	1.11
Upper myocardial T1 limits	1,293 ms	1,293 ms
Upper myocardial T2 limits	47 ms	47 ms
Upper myocardial ECV limits	28.5 %	28.5 %
Upper skeletal T1 limits	1,206 ms	1,206 ms
Upper skeletal ECV limits	14.9 %	14.9 %

*ECV, extracellular volume, LV, left ventricle, T1, longitudinal relaxation time, T2, transversal relaxation time*.

Normal values of LV ejection fraction, mass, volume, and mass/volume ratio, which are used to assess LV function and remodeling, and which are known to vary according to age and sex, were extracted from a local database of patients without any known cardiovascular disease (14). The final population was further selected according to age to provide a comparable mean and distribution to our COVID-19 population (for mean ± SD: 64.2 ± 7.5 vs. 64.2 ± 8.3 years). There were 74 men and 84 women with respective lower limits of 49% and 51% LV ejection fraction, and respective upper limits of 97 and 88 mL.m^−2^ for LV end-diastolic volume, 73 and 57 g.m^−2^ for LV mass, and 1.11 and 0.90 for LV mass/volume ratio. These limits are within the range of those already defined for ≥ 60 years old normal subjects in previously published CMR studies (18–20).

Normal T1 and ECV values were determined for both myocardium and shoulders skeletal muscles in a population extracted from a local database of patients who had been initially investigated for a mitral valve prolapse (21). The final selection only included patients without any complicated prolapse (absence of ≥ 2 mitral regurgitation, ventricular arrhythmias, or LV dysfunction) and without any other cardiovascular disease. This group included 30 subjects, 11 women and 19 men, with a mean age of 40 ± 18 years. The normal upper limits of T1 were computed as 1,293 ms for the myocardium and 1,206 ms for the skeletal shoulder muscles. For normal ECV, the respective upper limits were 28.5 and 14.9%. These limits are very similar to those obtained in previous CMR studies performed using a comparable methodology (22, 23).

For myocardial T2, we selected the threshold of 47 ms which corresponds to the upper limit of the 95% confidence interval observed in a study performed with a 3T magnet and with the same methodology as that used in our COVID-19 patients (24).

Late gadolinium enhancement was identified visually by a single observer (PM) as an increase in the signal from myocardial areas clearly distinct from the epicardial fat and cavitary blood. All transmural or sub-epicardial areas of LGE were considered as potentially related to myocarditis (14). This was not the case for the LGE evocative of a mid-wall septal fibrosis and commonly associated with LV hypertrophy and remodeling in the absence of any myocarditis (25).

### Statistical Analysis

Statistical analyses were obtained using the SPSS statistical software (IBM Statistics version 20). Qualitative variables were expressed as numbers and percentages and quantitative variables were expressed as medians with interquartile ranges. As the number of cases was not sufficiently large to assume a normal distribution, paired comparisons of quantitative variables between the two CMR visits were assessed using a non-parametric test: the Wilcoxon sum-rank test. Paired comparisons of qualitative variables were planned with Mc Nemar tests. *P* values were not not adjusted for possible multiple comparison effects given the exploratory nature of the present study, and *p* < 0.05 were considered to reflect significant differences.

## Results

Among the 222 COVID-19 patients hospitalized during the study period, 45 exhibited a peak troponin Ic > 50 ng/ml, and 19 fulfilled all study inclusion criteria. As detailed in Table 2, at the 1^st^ CMR, the median age was 66 [59–71] years, and the median from peak troponin was 3.2 [2.6–3.9] months. Seventeen patients (89%) were male, 11 (58%) had previously been identified with hypertension or obesity (6 with obesity and 8 with hypertension), and as many as 16 (84%) had been affected by an acute respiratory distress syndrome (ARDS) requiring mechanical ventilation at the acute phase.

**Table 2:** Main characteristics of the 19 patients with blood, clinical and CMR data collected on the day of the 1^st^ CMR, at a median of 3.2 months from the troponin peak.

**Age (years)**	**66 [59–71]**
Female	2 (11%)
Diabetes	7 (37%)
Dyslipidemia	6 (32%)
Hypertension	8 (42%)
Obesity (BMI > 30 kg/m^2^)	7 (37%)
ARDS at acute phase	16 (84%)
Peak troponin Ic at acute phase (ng/ml)	242 [83–896]
Delay time from peak Troponin (months)	3.2 [2.6–3.9]
Heart rate (bpm)	80.0 [64.8–82.1]
Systolic BP (mmHg)	134 [132–155]
Diastolic BP (mmHg)	81 [72–85]
End-diastolic LV volume (mL/m^2^)	63 [55–72]
LV ejection fraction (%)	58 [52–65]
LV mass (g/m^2^)	70 [59–80]
LV mass / volume ratio	1.20 [0.91–1.27]
End-diastolic RV volume (mL/m^2^)	56 [53–68]
RV ejection fraction (%)	55 [51–59]
Myocardial T1 (ms)	1,257 [1,221–1,270]
Myocardial T2 (ms)	38.0 [36.0–40.2]
Myocardial ECV (%)	27.6 [25.4–31.5]
Delayed retention myocarditis pattern	2 (11%)
Skeletal T1 (ms)	1,149 [1,110–1,149]
Skeletal ECV (%)	16.5 [14.4–22.4]
Hematocrit (%)	42.4 [40.3–43.9]
C Reactive Protein (mg/mL)	4 (4)
Troponin Ic (ng/ml)	6.0 [2.0–13.0]
Nt-pro BNP (pg/mL)	111 [36–259]
Albumin (g/L)	41.5 [39.1–46.4]
eGFR (ml/min/1.73 m^2^)	90 [84–90]

*ARDS, acute respiratory distress syndrome; BNP, brain natriuretic peptide; BMI, body mass index; BP, blood pressure; ECV, extracellular volume; eGFR, glomerular filtration rate estimated with the CKD-EPI formula and truncated at 90 ml/min/1.73 m^2^; LV, left ventricle; RV, right ventricle; T1, longitudinal relaxation time; T2, transversal relaxation time*.

### Baseline CMR

The 1^st^ CMR, recorded at a median 3.2 [interquartile range: 2.6–3.9] months from the troponin peak, showed a > 50% LV ejection fraction in all but 2 patients for whom it was only slightly lower (46% and 48%). However, as many as 12 (63%) exhibited LV concentric remodeling (i.e., high LV mass/volume ratio), which was associated with LV hypertrophy (i.e., high LV mass) in 9 cases. Myocardial tissue damage was documented in 11 patients (58%), including 9 increased myocardial ECVs, 3 abnormal T1, 1 abnormal T2, and 2 evocative late gadolinium enhancements (LGE). No pattern suggestive of myocardial infarction was observed (i.e., no sub-endocardial or transmural LGE).

For the shoulder skeletal muscles, abnormal values were observed for T1 in 2 cases (11%) and for ECV in 14 (74%). As detailed in Table 2, most plasma analytics were within normal or sub-normal concentration ranges (Table 2), including troponin Ic (all ≤ 29 ng/mL), CRP (all ≤ 10 mg/mL), NT-proBNP (all <450 pg/mL) and eGFR (all but one > 80 ml/min/1.73 m^2^).

### Follow-Up CMR

**Figure 2:**
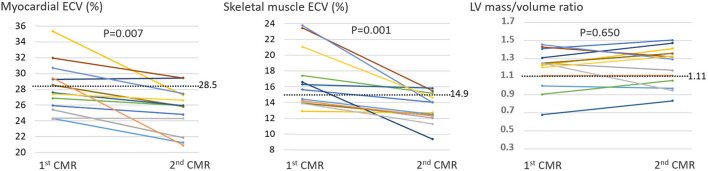
Individual variations between the 1^st^ and 2^nd^ CMR for the ECV from the myocardium and skeletal muscles, as well as for the LV mass/volume ratio, which was used to assess LV concentric remodeling and normalized to male values (i.e., with the values in women increased by 0.21 to compensate for the difference in the normal upper limits between women (0.90) and men (1.11)). The dashed lines represent the normal upper limits.

The 2^nd^ CMR was performed in 13 out of the 19 patients, at a median of 11.1 [11.0–11.7] months from peak troponin. LV function and remodeling parameters were unchanged between the 1^st^ and 2^nd^ CMR, but significant decreases in heart rate, myocardial T1, and particularly ECV from skeletal muscles and myocardium were observed (Table 3, Figure 2). Late contrast enhancement was still documented in 2 patients, and as evidenced in Figure 2, there was a return to normal or below the normal range for all ECVs of the myocardium and skeletal muscles.

**Table 3:** Changes in clinical, CMR and blood parameters of the 13 patients who underwent the two CMRs at medians of 3 and 11 months from peak troponin respectively.

	**1^**st**^ CMR**	**2^**nd**^ CMR**	***P*-value**
BMI (kg/m^2^)	27.4 [25.4–31.9]	30.9 [28.8–33.60]	0.103
Heart rate (bpm)	77.0 [65.4–82.2]	64.1 [57.5–79.3]	0.046
Systolic BP (mmHg)	134 [125–145]	142 [127–156]	0.173
Diastolic BP (mmHg)	81 [72–85]	81 [77–90]	0.166
End-diastolic LV volume	63 [53–71]	61 [51–69]	0.576
(mL/m^2^)			
LV ejection fraction (%)	60 [53–65]	56 [52–62]	0.388
LV mass (g/m^2^)	70 [60–82]	68 [59–82]	0.419
LV mass/volume ratio	1.23 [1.06–1.26]	1.12 [1.02–1.34]	0.650
End-diastolic RV volume	63 [53–71]	61 [51–69]	0.576
(mL/m^2^)			
RV ejection fraction (%)	60 [53–65]	56 [52–62]	0.388
Myocardial T1 (ms)	1,257 [1,225–12,646]	1,233 [1,192–1,256]	0.038
Myocardial T2 (ms)	37.6 [35.9–39.5]	38.0 [36.5–40.5]	0.576
Myocardial ECV (%)	27.4 [25.7–31.1]	25.9 [23.1–27.3]	0.007
Delayed retention	2 (14%)	2 (14%)	1.000
myocarditis pattern			
Skeletal T1 (ms)	1,122 [1,104–1,173]	1,134 [1,104–1,228]	0.382
Skeletal ECV (%)	15.6 [14.2–19.2]	12.7 [12.2–14.9]	0.001
Hematocrit (%)	42.2 [40.1–43.4]	42.4 [39.2–44.5]	1.000
C Reactive Protein (mg/mL)	4 (4)	4 [4–9.3]	0.028
Troponin Ic (ng/ml)	5.5 [2.0–13.2]	5.0 [2.75–12.25]	0.893
Nt-pro BNP (pg/mL)	111 [41–133]	56 [35–52]	0.285
Albumin (g/L)	42.0 [40.3–47.4]	44.1 [42.7–46.0]	0.388
eGFR (ml/min/1.73 m^2^)	90 [87–90]	90 [82–90]	0.221

*BMI, body mass index; BNP, brain natriuretic peptide; BP, blood pressure; ECV, extracellular volume; eGFR, glomerular filtration rate estimated with the CKD-EPI formula and truncated at 90 ml/min/1.73 m^2^; LV, left ventricle; RV, right ventricle; T1, longitudinal relaxation time; T2, transversal relaxation time*.

## Discussion

**Figure 3:**
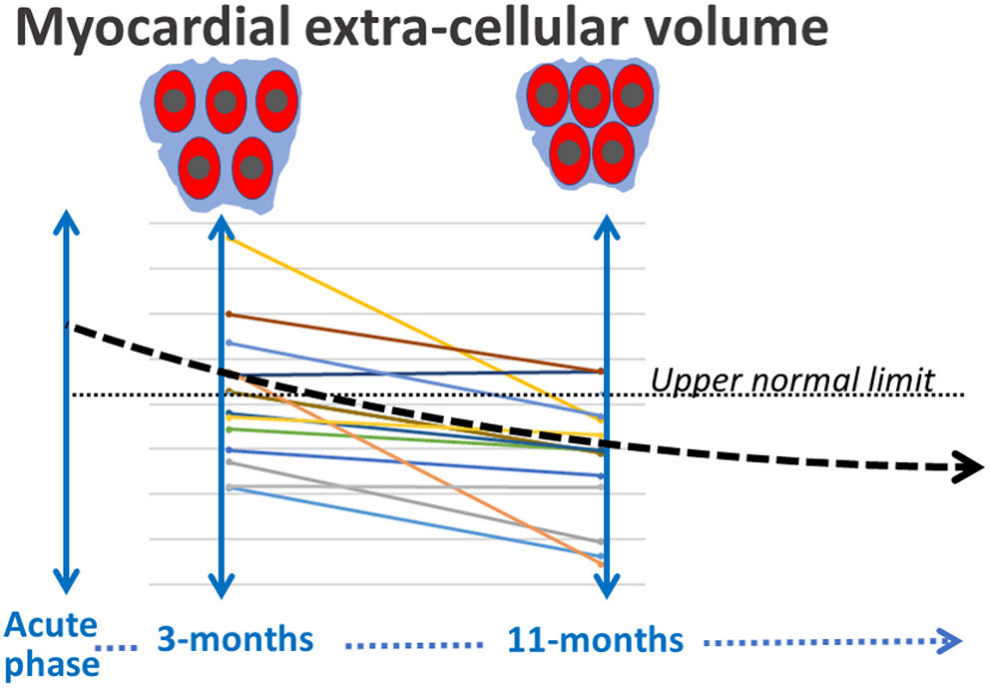
Schematic representation of the progressive decrease during the first year of the myocardial ECV, presumably in line with a delayed resolution of the interstitial edema.

As illustrated by a schematic representation in Figure 3, a frequent increase in ECV affecting the myocardium and skeletal muscles and which regressed during the first year, constituted the main observation in our consecutive series of patients with no prior history of cardiac disease but for whom COVID-19 induced myocardial damage was ascertained by a significant rise in blood troponin Ic. A COVID-19 etiology of these increased ECV values is supported by their return to normal or below the normal range at 1 year (Figure 2), in contrast to the stability observed for other CMR parameters. This ECV evolution likely reflects the resolution of the extracellular interstitial edema, which is commonly observed in the heart and other organs in COVID-19 autopsy studies (26, 27).

CMR already documented an increased myocardial ECV at the acute or sub-acute phase of COVID-19 and associated with increases in myocardial T1 and T2 (5, 6). This observation was attributed to inflammatory edema at the time. Our serial CMR data show that this increased ECV: (i) also affects skeletal muscles, (ii) resolves very progressively, given its common persistence at a median of 3 months from the acute phase (i.e., at the time of the 1^st^ CMR), and (iii) is then no longer associated with any evident signs of active damage or inflammation (i.e., based on the normal or below the normal range of CRP, troponin and myocardial T2 values).

Interestingly, the decrease in ECV at 1 year was observed in our COVID patients irrespective of the presence or absence of an abnormal ECV at 3-months (see Figure 2). This decrease might thus be commonly involved in the recovery of severe COVID-19. In addition, a possible role of a non-specific response associated with ARDS needs further investigation. Indeed, 84% of our COVID-19 patients presented an ARDS at the acute phase, and ARDS patients are commonly affected by muscle dysfunction in both early and late stages, constituting a significant morbidity factor (28, 29).

This increased ECV was not associated with any evident deterioration of cardiac function, with LV ejection fractions and volumes remaining normal or below the normal range during follow-up according to the CMR, as well as the echocardiography data (see Supplementary Data File). As many as 63% of our patients were affected by LV concentric remodeling, an indicator of increased cardiovascular risk (15, 16). However, this remodeling was unchanged between the two evaluations, and it may constitute an underlying pathology due to the risk factors shared by concentric remodeling and severe COVID-19 (age, obesity, hypertension).

A main limitation is the small sample size of the present study population, and further studies will be required to confirm the results.

### Conclusion and Perspectives

The present serial CMR study shows a slow return to normal of the extracellular volume of the myocardium and skeletal muscles in many patients with no history of cardiac disease, but for whom an increase in blood troponin-Ic ascertained COVID-19 induced myocardial damage. This observation is likely due to a delayed resolution of the interstitial edema, which is known to affect severe COVID-19 patients. Associations with long-COVID symptoms (30) need to be investigated on a larger scale. This might help to better understand and perhaps to prevent or treat these symptoms. The potential role of a non-specific response associated with ARDS also requires further investigation.

## Data Availability Statement

The raw data supporting the conclusions of this article will be made available by the authors, without undue reservation.

## Ethics Statement

The studies involving human participants were reviewed and approved by Ethics Committee of the Nancy University Hospital. Written informed consent for participation was not required for this study in accordance with the national legislation and the institutional requirements.

## Author Contributions

Seven authors contributed significantly to the analysis and interpretation of the data (LF, M-SR, NJ, J-SL, GH, OH, and P-YM), and/or to the writing or revision of the manuscript (LF, NP, J-SL, OH, and P-YM), the four others collaborated in the study implementation, and/or management of the included subjects (DM, FG, NP, and CS-S). All authors contributed to the article and approved the submitted version.

## Funding

This study was funded by CHRU-Nancy, Nancy, France. Hospital's own funds were used for the study organization.

## Conflict of Interest

The authors declare that the research was conducted in the absence of any commercial or financial relationships that could be construed as a potential conflict of interest.

## Publisher's Note

All claims expressed in this article are solely those of the authors and do not necessarily represent those of their affiliated organizations, or those of the publisher, the editors and the reviewers. Any product that may be evaluated in this article, or claim that may be made by its manufacturer, is not guaranteed or endorsed by the publisher.
