# Supplementary material for: Long-Lasting Myocardial and Skeletal Muscle Damage Evidenced by Serial CMR During the First Year in COVID-19 Patients From the First Wave
Source: Front Cardiovasc Med. 2022 Mar 9;9:831580. doi: 10.3389/fcvm.2022.831580 (PMC8959613; doi:10.3389/fcvm.2022.831580)
Supplement: Supplementary file 1 [file Table_1.DOCX]

**Echocardiography Results**

A complete set of echocardiography data was obtained, on the day of the 1^st^ CMR, for 16 of the 19 included patients. LV ejection fraction, assessed by a biplane automatic method, and LV end-diastolic diameter and LV mass, were always within the normal ranges, except in one case of LV hypertrophy (a man with an LV mass > 115 g/m^2^). In contrast, the relative wall thickness was greater than 0.45, consistent with definite LV concentric remodeling, in 6 among the 16 patients (38%).

Twelve patients also had a complete set of echocardiography data on the day of the 2^nd^ CMR. In agreement to what was observed by CMR, none of the echocardiography/Doppler data showed any significant changes in the systolic function of LV (ejection fraction) or of the right ventricle (TAPSE) and also not in the Doppler parameters impacted by the diastolic function.

**Supplementary Table:** Main echography and Doppler parameters used to assess cardiac function and remodeling on the day of the 1^st^ CMR in the overall echography group (n=16), and their evolution between the 1^st^ and 2^nd^ CMR in the follow-up echography group (n=12).

|  | **Overall** | **Follow-up** | | |
| --- | --- | --- | --- | --- |
|  | *1^st^ CMR day* | *1^st^ CMR day* | *2^nd^ CMR day* | *P-value* |
| LV ejection fraction (%) | 60 [57-65] | 61 [58-65] | 61 [57-64] | 0.535 |
| LV end-diastolic diameter (mm) | 46.9 [41.5-53.5] | 45.4 [40.3-52.8] | 47.5 [40.3-53.0] | 0.824 |
| LV mass (g/m^2^) | 94 [67-109] | 84 [65-105] | 87 [68-111] | 0.722 |
| Relative wall thickness | 0.39 [0.29-0.49] | 0.44 [0.34-0.52] | 0.43 [0.33-0.51] | 0.529 |
| TAPSE | 23 [18-24] | 23 [19-24] | 21 [19-26] | 0.958 |
| Left atrial volume (ml/m^2^) | 26.7 [21.3-26.7] | 26.7 [22.3-31.3] | 29.5 [24.9-45.0] | 0.071 |
| E/A ratio | 0.80 [0.69-1.04] | 0.80 [0.66-1.04] | 0.85 [0.59-1.11] | 0.480 |
| Lateral e' velocity (m/s) | 0.09 [0.07-0.11] | 0.09 [0.07-0.11] | 0.11 [0.07-0.13] | 0.103 |
| Lateral E/e' ratio | 9.1 [6.3-10.0] | 9.1 [6.3-9.7] | 6.9 [4.9-9.4] | 0.182 |

LV: left ventricle, TAPSE: tricuspid annular plane systolic excursion function (E/A ratio, lateral e' velocity, lateral E/e' ratio).
